# Performance of machine learning models in estimation of ground reaction forces during balance exergaming

**DOI:** 10.1186/s12984-022-00998-5

**Published:** 2022-02-13

**Authors:** Elise Klæbo Vonstad, Kerstin Bach, Beatrix Vereijken, Xiaomeng Su, Jan Harald Nilsen

**Affiliations:** 1 Department of Computer Science, Norwegian University of Science and Technology, Trondheim, Norway; 2 Department of Neuromedicine and Movement Science, Norwegian University of Science and Technology, Trondheim, Norway

**Keywords:** Weight shifting, Balance training, Exergaming, Ground reaction force, Deep learning, Long short-term memory networks, XGBoost

## Abstract

**Background:**

Balance training exercise games (exergames) are a promising tool for reducing fall risk in elderly. Exergames can be used for in-home guided exercise, which greatly increases availability and facilitates independence. Providing biofeedback on weight-shifting during in-home balance exercise improves exercise efficiency, but suitable equipment for measuring weight-shifting is lacking. Exergames often use kinematic data as input for game control. Being able to useg such data to estimate weight-shifting would be a great advantage. Machine learning (ML) models have been shown to perform well in weight-shifting estimation in other settings. Therefore, the aim of this study was to investigate the performance of ML models in estimation of weight-shifting during exergaming using kinematic data.

**Methods:**

Twelve healthy older adults (mean age 72 (± 4.2), 10 F) played a custom exergame that required repeated weight-shifts. Full-body 3D motion capture (3DMoCap) data and standard 2D digital video (2D-DV) was recorded. Weight shifting was directly measured by 3D ground reaction forces (GRF) from force plates, and estimated using a linear regression model, a long-short term memory (LSTM) model and a decision tree model (XGBoost). Performance was evaluated using coefficient of determination ($R^2$) and root mean square error (RMSE).

**Results:**

Results from estimation of GRF components using 3DMoCap data show a mean (± 1SD) RMSE (% total body weight, BW) of the vertical GRF component ($F_z$) of 4.3 (2.5), 11.1 (4.5), and 11.0 (4.7) for LSTM, XGBoost and LinReg, respectively. Using 2D-DV data, LSTM and XGBoost achieve mean RMSE (± 1SD) in $F_z$ estimation of 10.7 (9.0) %BW and 19.8 (6.4) %BW, respectively. $R^2$ was $>.97$ for the LSTM in the $F_z$ component using 3DMoCap data, and $>.77$ using 2D-DV data. For XGBoost, $F_z$ $R^2$ was $>.86$ using 3DMoCap data, and $>.56$ using 2D-DV data.

**Conclusion:**

This study demonstrates that an LSTM model can estimate 3-dimensional GRF components using 2D kinematic data extracted from standard 2D digital video cameras. The $F_z$ component is estimated more accurately than $F_y$ and $F_x$ components, especially when using 2D-DV data. Weight-shifting performance during exergaming can thus be extracted using kinematic data only, which can enable effective independent in-home balance exergaming.

## Background

Being able to maintain or regain balance is a cornerstone for sustained independence in daily life of older adults. Balance, or postural control, is a complex motor skill that depends on the coordination and function of multiple bodily systems [1]. As we age, our postural control deteriorates gradually, increasing the risk of falls and decreasing community mobility and quality of life. These are major factors of increased risk of disability and mortality in elderly [2]. Targeted balance exercise improves postural control, and exercises typically included in exercise programs for balance training are for example leaning, reaching, and weight shifting [3]. These types of exercises have been shown to reduce fall risk [4, 5] by improving dynamic stability during gait [6] as well as anticipatory and reactive balance ability [3]. Research has shown that technological tools that provide visual biofeedback and guidance can improve the potential effect of such exercises [7, 8]. By using exercise games (so-called exergames), biofeedback can be provided in a motivational and fun manner [9, 10]. In weight-shifting exercises, biofeedback is provided typically by using force-sensing equipment placed under the person’s feet or inside the shoes. One of the most accurate types of force measurement equipment are piezoelectric force plates [11]. These return three-dimensional ground reaction force (GRF) vectors, which are precise representations of the magnitude and directions of the force exerted on the plates by the person’s feet.

Even though force plates are effective to provide biofeedback in balance exercising, they are rarely used outside laboratory settings as they are very costly and resource-demanding to use. More user-friendly substitutes, such as the Wii Balance Board (Nintendo Co Ltd, Japan) have been developed and are used in exergames for balance training. These, however, have drawbacks in settings other than casual gaming, as they are less accurate and register limited information only [12, 13]. They have also been reported to cause uncomfortable and unsafe experiences, and increase fear of falling (see, e.g. [14]). This makes the Wii Balance Board less suited for in-home use by elderly persons. More recent exergames for balance training started using kinematic data from depth-sensing cameras such as the Kinect (Microsoft Inc). However, using kinematic data as a proxy for kinetic information is problematic due to insufficient accuracy in the kinematic data provided [15]. Accurate and useful information about exercise performance is vital if independent exercise in older adults is to be effective. At the same time the equipment necessary to provide this information has to be easy to use and resource-friendly, without sacrificing accuracy.

We know from previous research that GRF can be successfully estimated in other movements using machine learning (ML) methods. In [16], GRF was estimated during gait using a long-short term memory (LSTM) model, achieving estimates of GRF components within 12% RSME. In [17, 18], feed-forward artificial neural networks (ANN) gave an RMSE of GRF forces of $< 10$% in all three components during gait and asymmetric movements. Additional studies successfully estimated GRF during running [19] and activities of daily living [20]. These studies base their estimation on a biomechanical model computed from a 3DMoCap system, which requires measuring several points on the body over time using e.g. inertial measurement sensors [21]. Furthermore, this approach also requires physical measurements of the body of the person playing to scale the biomechanical model. This, combined with an additional computational layer for the calculation of the biomechanical model and the required practical procedures (e.g., full-body device placement), makes it an implausible method for use in in-home settings for elderly users, or outside of a laboratory in general [22].

Nonetheless, the direction of using LSTM does seem promising. LSTM is a form of neural network where sequential data is processed recurrently and important features are “remembered” for future predictions/estimations [23]. LSTMs are also relatively quick in estimation, allowing for real-time estimates which is a requirement when giving feedback during exergaming. Another approach, widely used because of its powerful method of representing the relationships in the data, is decision tree-based methods. Recently, a version of decision trees, called “extremely boosted gradient trees” (XGBoost, [24]), has been shown to outperform other regression methods [25], including in estimation of forces in a biomechanical setting [26]. In addition, decision trees are inherently transparent in their decision making process, which is a highly valuable feature. This can provide information about which joints are important in estimating GRF, which might inform decisions on relevant motion tracking tools in this context.

Furthermore, it was recently shown that standard digital 2D video can be used to extract 2D kinematic data of joint positions (e.g. [27–29]). This makes it possible to use devices such as smartphones, tablets, or web cameras to capture movements. We propose utilizing positional data of joint centers from pose estimation systems in combination with machine learning methods to estimate 3D GRF components during balance exergaming. This would remove the need for any physical measurements or biomechanical model of the person playing, and achieving this using a standard digital video camera only would make the system very easy to use and suitable for in-home guided exercise. Therefore, the aim of this paper is two-fold: (1) to investigate the performance of an LSTM model and an XGBoost model for estimation of ground reaction forces during balance exergaming, and (2) to compare performance between using 3D and 2D kinematic data.

## Methods

### Participants and protocol

Twelve healthy older adults were recruited from local exercise groups. Mean age was $72 \pm 4.2$ years, ten were female. Exclusion criteria were physical or cognitive injuries/impairments that affected their balance and gait ability, and age $< 50$ or age $> 80$ years. Data was collected at the Movement Capture and Visualization Laboratory at the Norwegian University of Science and Technology in Trondheim, Norway in June 2019.

### The exergame

**Fig. 1:**
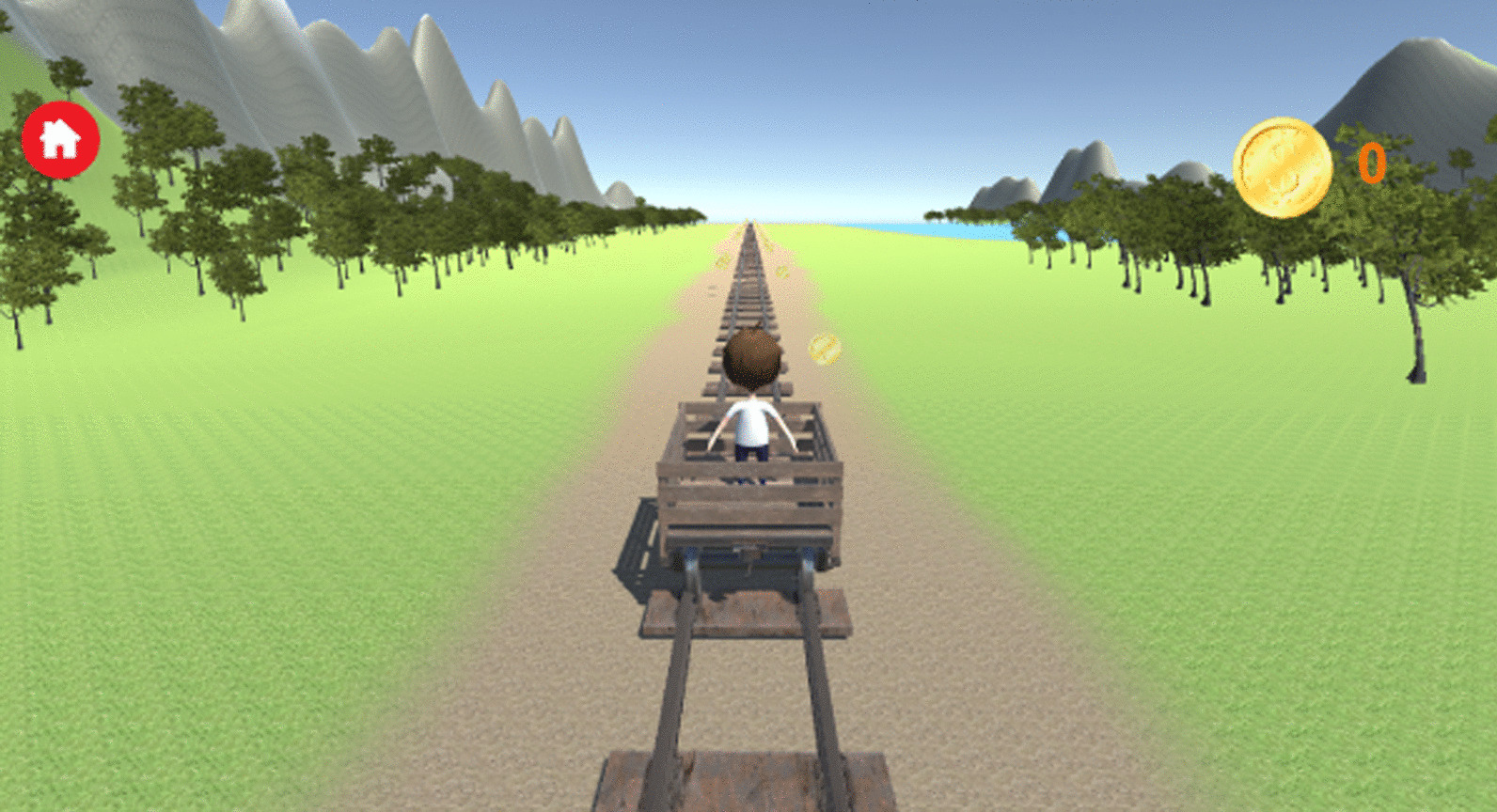
Game interface

**Fig. 2:**
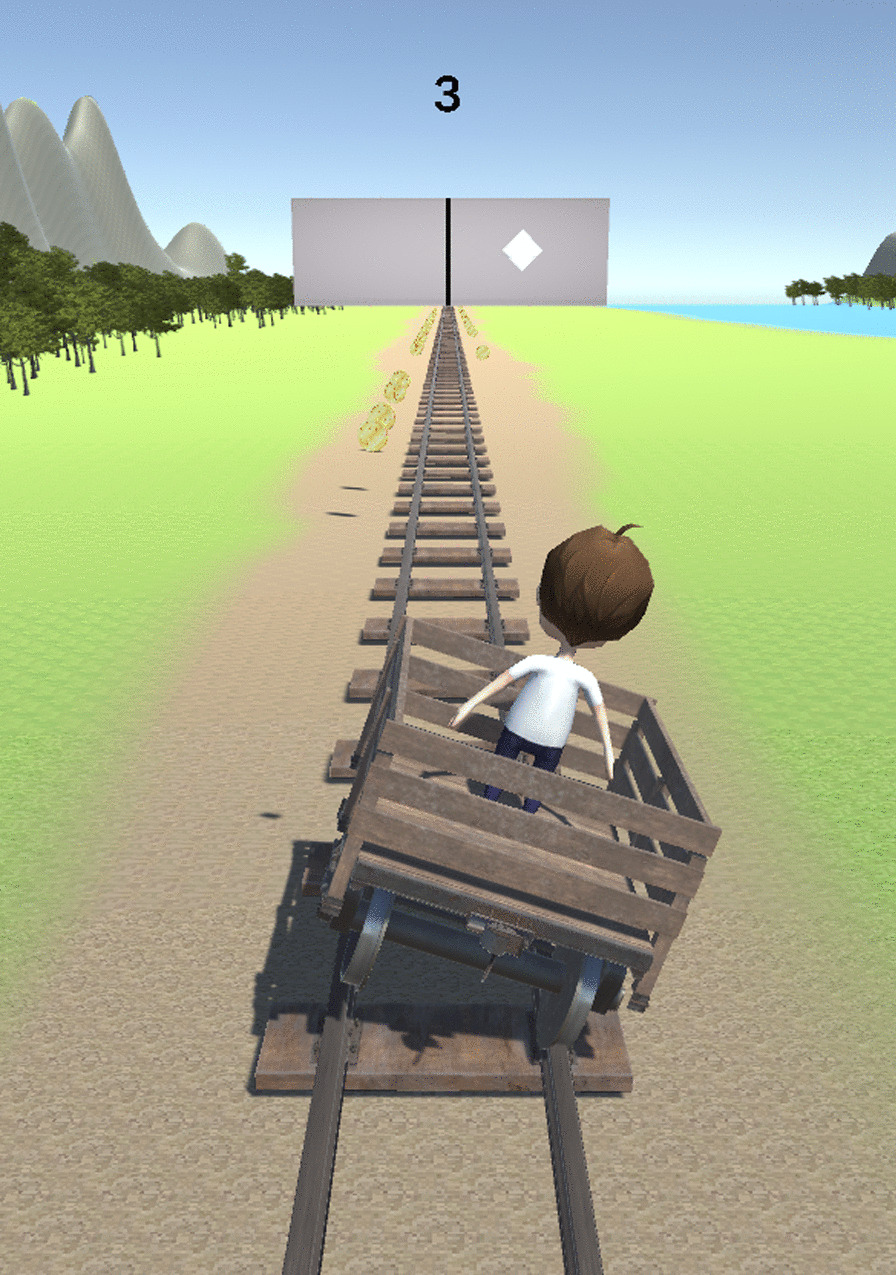
Cart tilting sideways to hit coin

A custom exergame for balance training was used in this study, using Kinect (v2, Microsoft Inc) to track participants’ movements for input to the game. The exergame was designed to elicit medio-lateral weight shifts from the user: An avatar representing the user was shown in a rail cart on a train rail, as seen in Fig. 1. Along each side of the rail there were coins that the user should try to hit by tilting the cart sideways, which was achieved by shifting their body weight over to the foot that on the side of the coin (Fig. 2). There were never more than two coins consecutively on one side. There were approximately 100 coins in total, with 50 % appearing on each side.

### Equipment

**Fig. 3:**
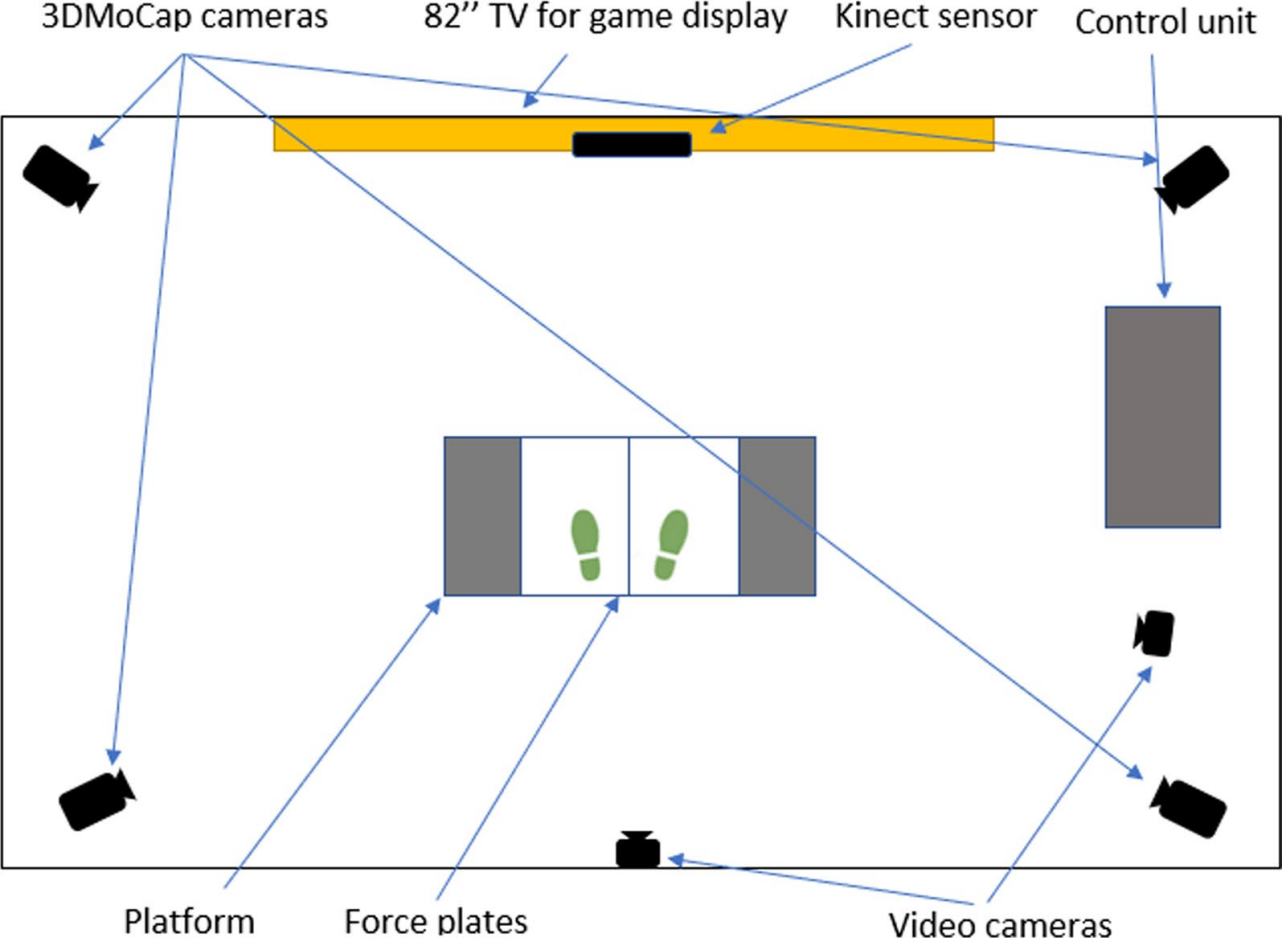
Experimental setup

A four-camera (MX400, 90 Hz, Qualisys Inc, Sweden) setup was used for capturing 3D motion data (3DMoCap) from participants. The Plug-in-Gait Full Body (PiG-FB, [30]) marker setup, excluding head and hands, was used. Two digital cameras (GoPro Hero Black 3+, 25 Hz, GoPro Inc) placed 200 cm behind and to the side of the player were used to capture player movements simultaneously with the 3DMoCap system. To capture force data, two force plates (60 × 5 × 40 cm, 1000 Hz, Kistler AB) were used, one under each foot of the player. The experimental setup can be seen in Fig. 3.

### Preprocessing

To extract joint center positions from 2D-DV data, the DeepLabCut(DLC, [28]) framework was used. The 3DMoCap data was gap-filled and the joint center positions were extracted using the standardized PiG-FB biomechanical model implemented in Nexus (v. 2.9, Vicon Motion Systems Ltd). The joint center positions extracted from both data sources were ankles, knees, hips, shoulders, elbows and wrists. From the 3DMoCap system the anterio-posterior (X), medio-lateral (Y) and vertical (Z) positions relative to the Qualisys global coordinate system origin were extracted, and in the 2D-DV data the vertical (Y) and medio-lateral (X) positions relative to the 2D-DV camera origin were extracted. This resulted in 36 input features from the 3DMoCap system, and 24 features from the 2D-DV system. The data was then normalized to the [0,1] range. Data was synchronized by resampling joint center data from digital video using the 3DMoCap data frequency as reference. Force components $F_x$ (anterio-posterior), $F_y$ (medio-lateral) and $F_z$ (vertical) were extracted from the force plate data. GRF components were scaled to body weight (BW) for each time frame. The video data of ankles was occluded in participants 4, 8, 9, and 10, resulting in missing ankle data for these participants. 3DMoCap data from participants 1 and 2 was corrupted, and not used in further analyses.

### Machine learning models

Python v. 3.7.10 was used for all analyses and evaluation. Sci-Kit Learn [31] was used for multivariate linear regression (LinReg), GridSearchCV and feature importance, and for evaluation of model performances; the Keras framework [32] was used to build the LSTM model; and XGBoost was implemented using the XGBoost package for Python (https://github.com/dmlc/xgboost).

**Fig. 4:**
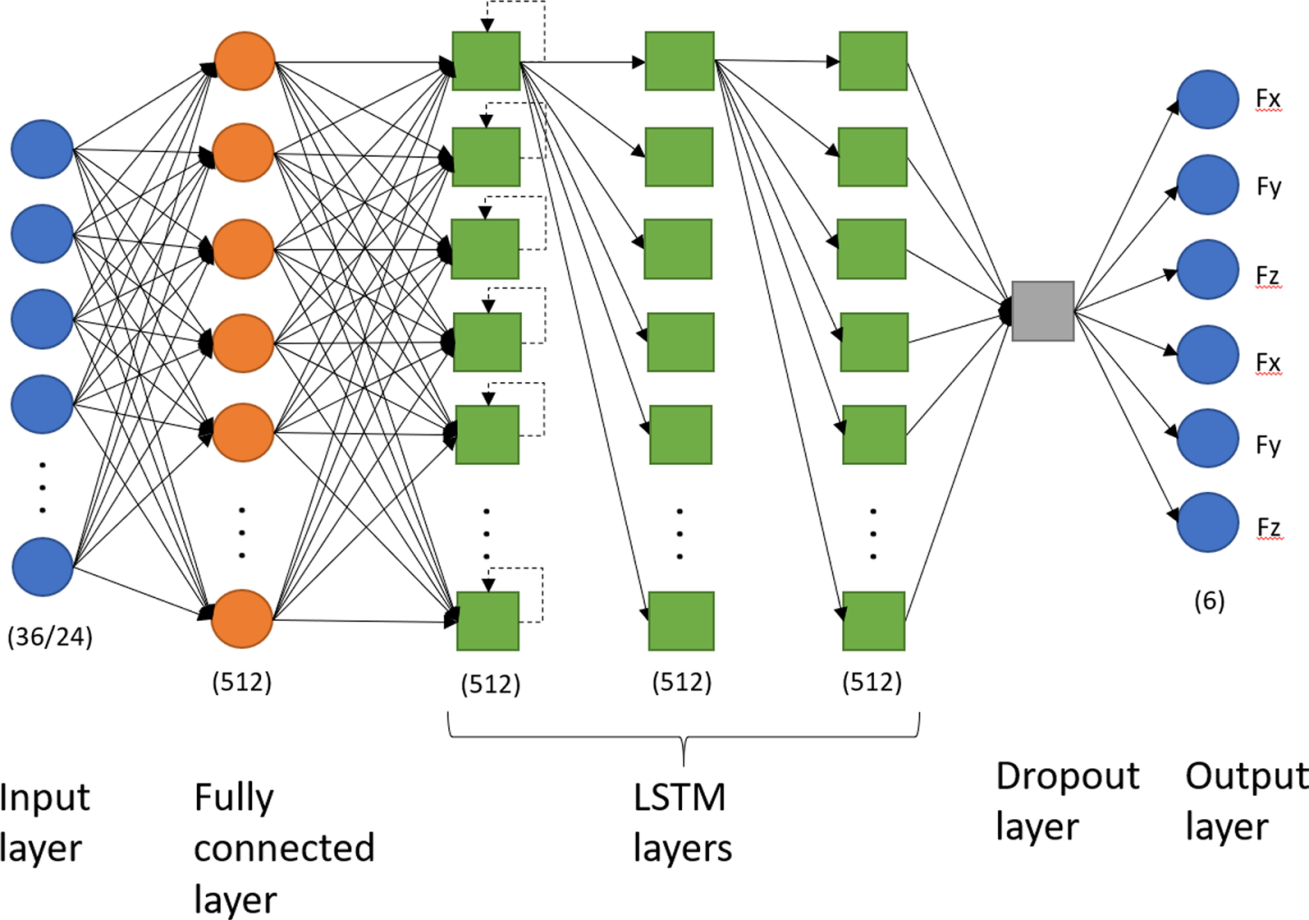
Schematic of the stacked long-short term memory (LSTM) model we implemented. For clarity, not all connections are shown; all layers are fully connected, and all LSTM units have the recurrent connection depicted in the first LSTM layer. The input layer consists of 36 nodes for 3DMoCap data and 24 nodes for 2D-DV data before feature selection

Multivariate linear regression (LinReg) was used as a baseline model for reference purposes. XGBoost is an improved version of decision tree models that combines a random forest technique of feature bagging, and a gradient decent method to reduce boosting error—hence the name “gradient boosting”. This has been shown to perform well on a wide range of non-linear estimation tasks [24]. Long short-term memory model (LSTM) is a version of a recurrent neural network. Stacked LSTM is a version of LSTM models that utilizes several layers of LSTM nodes, which has been shown to improve performance over single layer LSTMs [33]. A schematic of the stacked LSTM model we implemented in this study can be seen in Fig. 4. There is one dense input layer, three hidden layers of 512 nodes each, a dropout layer (.2), and a dense output layer of 6 nodes with sigmoid activation: one for each dimension in the force data for each force plate.

### Parameters and optimization

Hyperparameters for the XGBoost model were tuned using GridSearchCV with five cross-validation iterations, and the most optimal hyperparameter settings were found. The hyperparameter grid searched can be found in Table 1. The hyperparameter values in bold font were the ones found to yield the highest performance, and were used in training the final XGBoost model.

Optimization of the LSTM network was conducted using Adam optimizer [34] with an initial learning rate of .0001, decay steps 10,000, and decay rate .96. The model was trained for 200 epochs, with a minimum rate of improvement of loss (mean squared error, MSE) of .0003 for three consecutive epochs.

**Table 1:** Hyperparameter space searched in GridSearchCV for the XGBoost model after feature selection

Hyperparameter	Values searched
Learning rate	.001, **.005**, .01, .05, .10, .15
Max depth	5, 7, 9, **12**, 15
No. Estimators	50, 100, **200**, 500, 700
Min. child weight	1, 3, **5**, 7
Gamma	.0, **.1**, .2

Values in **bold** were used in further analyses

A leave-one-group-out cross validation was performed on all models, where one group was the data from one participant, which served as the test set in each iteration. This was performed on the joint data from 3DMoCap and 2D-DV systems. For evaluation, mean of left and right foot (1SD) root mean square error (RMSE), and mean (1SD) coefficient of determination ($R^2$) for the different cross-validation splits was computed.

## Results

The results showing feature selection and subsequent estimation performance of LSTM, XGBoost and LinReg using 3DMoCap and 2D-DV data, are presented as RMSE in Table 2 and $R^2$ in Fig. 7. Figure 8 shows illustrative example graphs of estimation performance of the three models using 3D and 2D data, over a randomly selected sequence (1000 frames) from one person during one trial of play.

Furthermore, the contribution of each joint center to estimation performance was computed using a permutation procedure. Here, the data in each feature is shuffled in a random manner, which breaks the real-world relationship between the feature and the target. The resulting difference in estimation performance between using the shuffled and un-shuffled feature is indicative of how much the model depends on this feature [35]. This is then repeated for all features, and inform about which features, i.e. joint centers, are most important to the estimation performance. Results from the feature importance analysis, using 3DMoCap data, showed that eight joint centers contributed with 82.9% of the information needed to estimate GRF components. These joint centers were right and left wrist, right elbow, left knee, and torso joint centers (left and right shoulders, and left and right hip joints). The models were subsequently retrained using these joints.

**Fig. 5:**
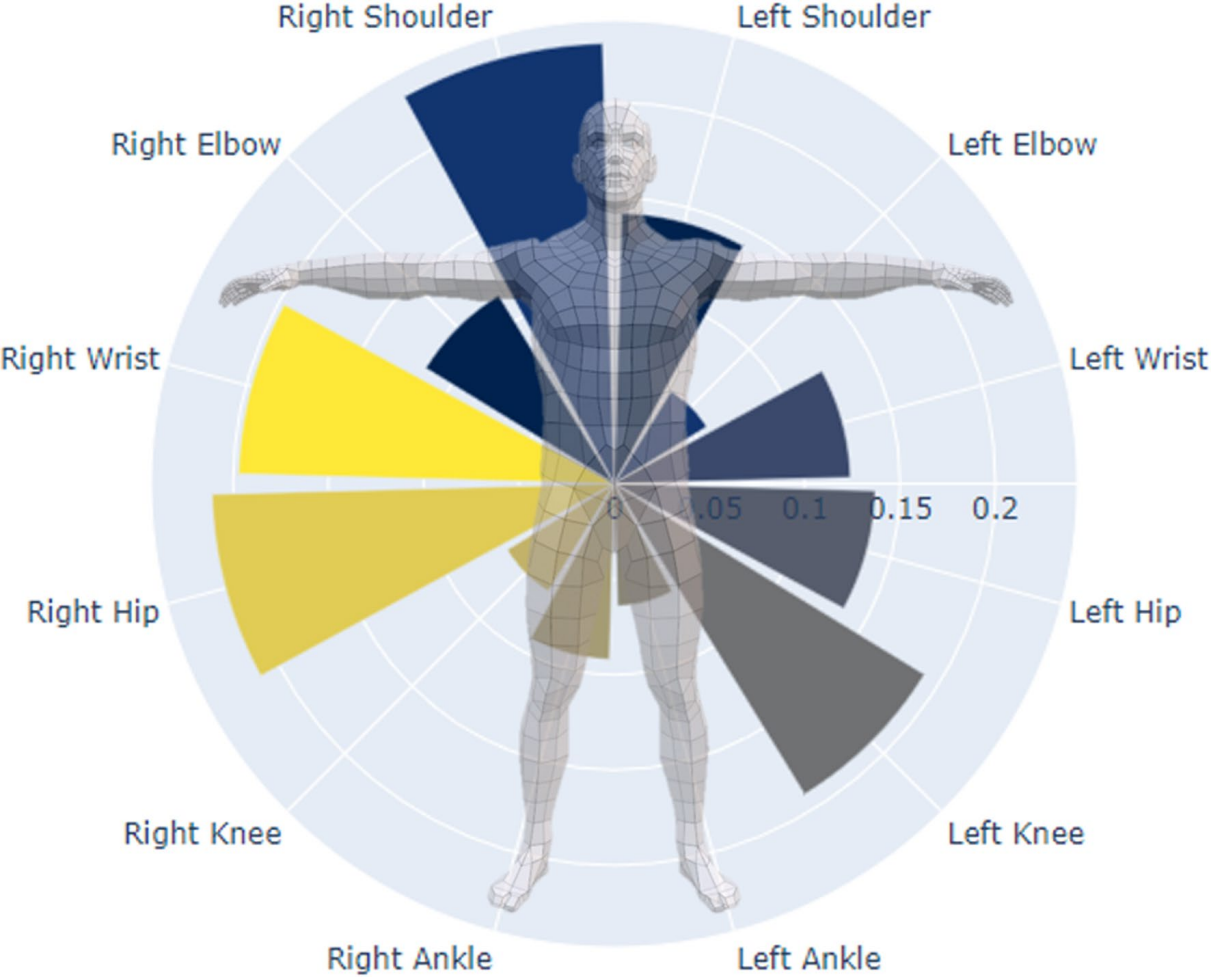
Overview of the joint centers’ total impact (fraction of $R^2$) on estimation performance when using 3DMoCap data

**Fig. 6:**
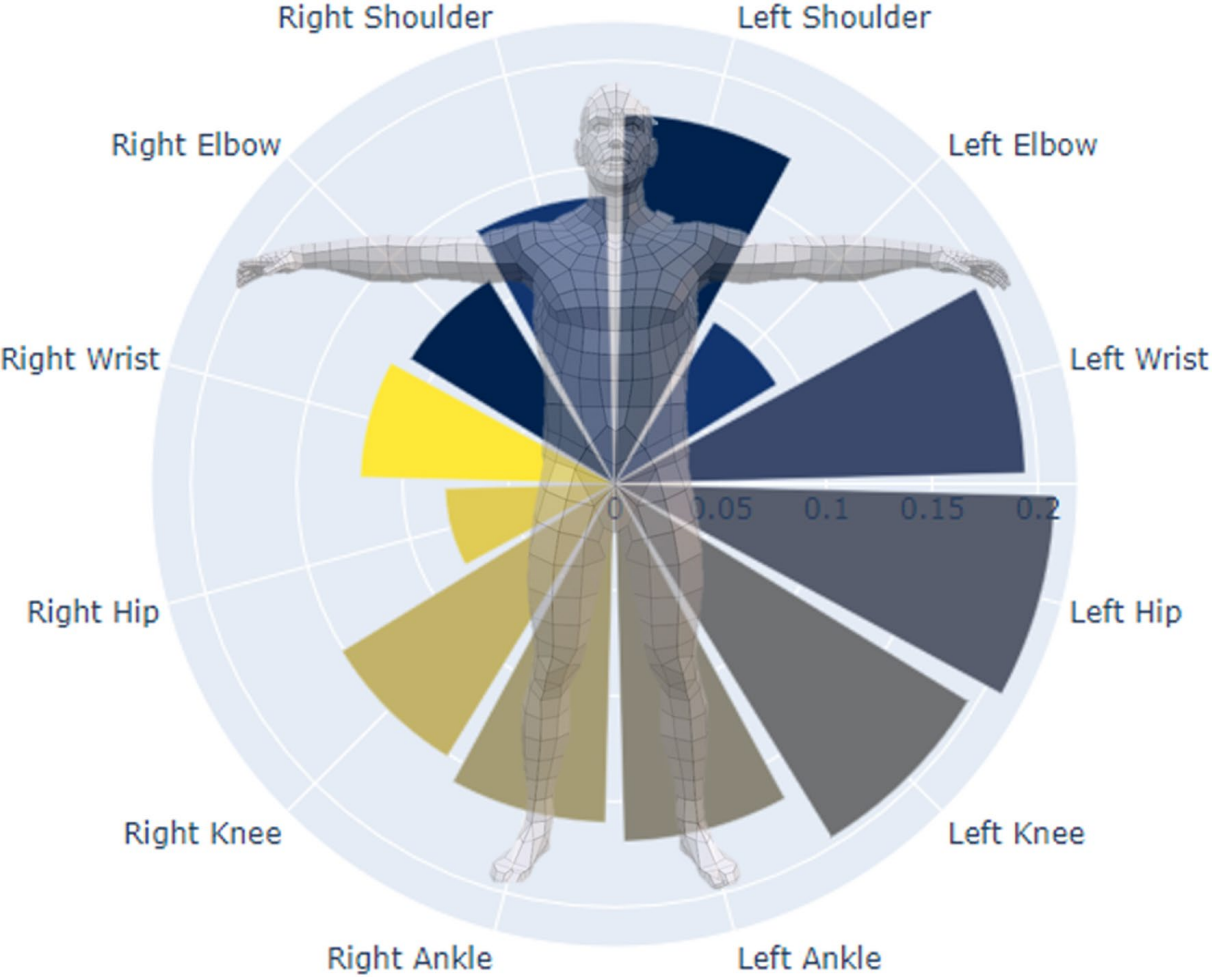
Overview of the joint centers’ total impact (fraction of $R^2$) on estimation performance when using 2D-DV data

Using 2D-DV data, there were also eight joint centers that had a total contribution of 78%: Left wrist, shoulder, hip, knee and ankle, and right shoulder, knee, and ankle. The relative contributions of all joint centers can be seen in Figs. 5 and 6.

### Estimation error

Prediction performance is presented in Table 2, with the mean (± 1SD) RMSE (% BW) for the three models using 3DMoCap and 2D-DV data for the three force components. The LSTM model outperforms both XGBoost and LinReg when using both 3DMoCap and 2D-DV data. The XGBoost model achieves at the same level as LinReg using both 3DMoCap and 2D-DV data. Lowest mean RMSE (4.3% BW) was achieved by the LSTM model on the $F_z$ component using 3DMoCap data; highest (23.5% BW) was the LinReg model in the $F_y$ component using 2D-DV data. RMSE was generally higher using 2D-DV data than when using 3DMoCap data.

**Table 2:** Mean (± 1SD) RMSE (% BW) achieved by the three models from estimation of all three components of GRF

	3DMoCap			2D-DV		
	LSTM	XGBoost	LinReg	LSTM	XGBoost	LinReg
$F_x$	10.3 (6.2)	17.3 (3.6)	17.6 (3.8)	12.7 (7.6)	18.6 (3.8)	18.2 (3.8)
$F_y$	7.1 (4.6)	13.4 (3.9)	19.0 (2.7)	10.4 (6.0)	20.2 (4.0)	23.5 (8.5)
$F_z$	4.3 (2.5)	11.1 (4.5)	11.0 (4.7)	10.7 (9.0)	19.8 (6.4)	18.6 (6.4)

### Model fit

**Fig. 7:**
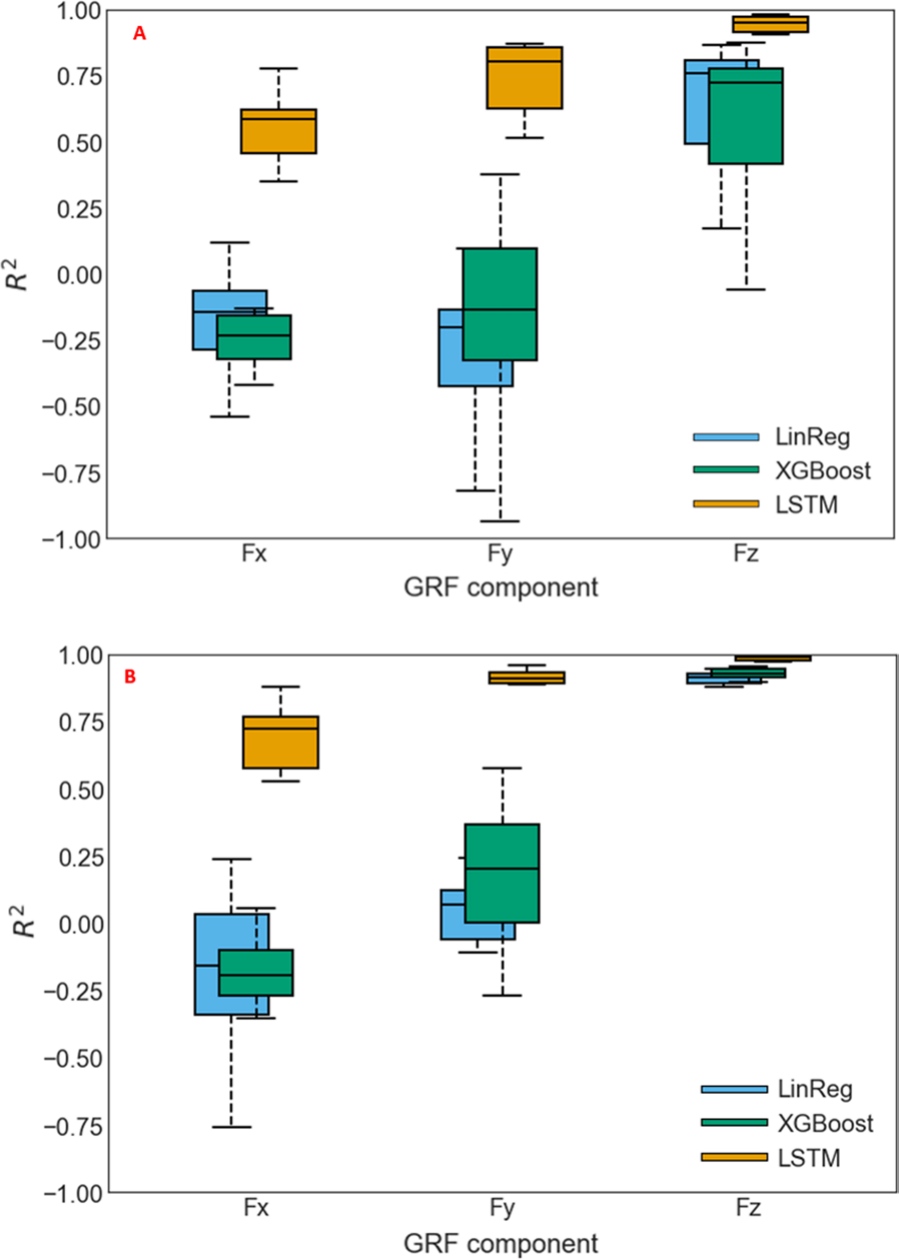
Box plots showing median $R^2$ from LSTM, XGBoost, and LinReg models in all three GRF components. **A** Results using 2D-DV data, and **B** MoCap data

As shown in Fig. 7, the LSTM $R^2$ is consistently higher than in the XGBoost and LinReg model using both MoCap and 2D-DV data. Using the MoCap data, the mean (± 1SD) LSTM $R^2$ was .589 (.34), .796 (.31), and .971 (.05) in the $F_x$, $F_y$, and $F_z$ components, respectively, and XGBoost $R^2$ was − .246 (.27), .114 (.36), and .863 (.16), respectively. The LinReg model achieved a mean $R^2$ of − .168 (.28), − .054 (.21), and .856 (.17), respectively. Using 2D-DV data, all models achieved slightly lower $R^2$. LSTM achieved mean (± 1SD) $R^2$ of .379 (.55) in $F_x$, .579 (.58) in $F_y$ and .770 (.45) in $F_z$. XGBoost mean (± 1SD) $R^2$ in $F_x$ was − .313 (.26), − .234 (.53) in $F_y$, and .564 .(.31) in $F_z$. Here, the LinReg results were mean (± 1SD) $R^2$ of − .266 (.39), − .950 (2.23), and .617 (.28) for the $F_x$, $F_y$, and $F_z$ components, respectively.

### Estimation plots

**Fig. 8:**
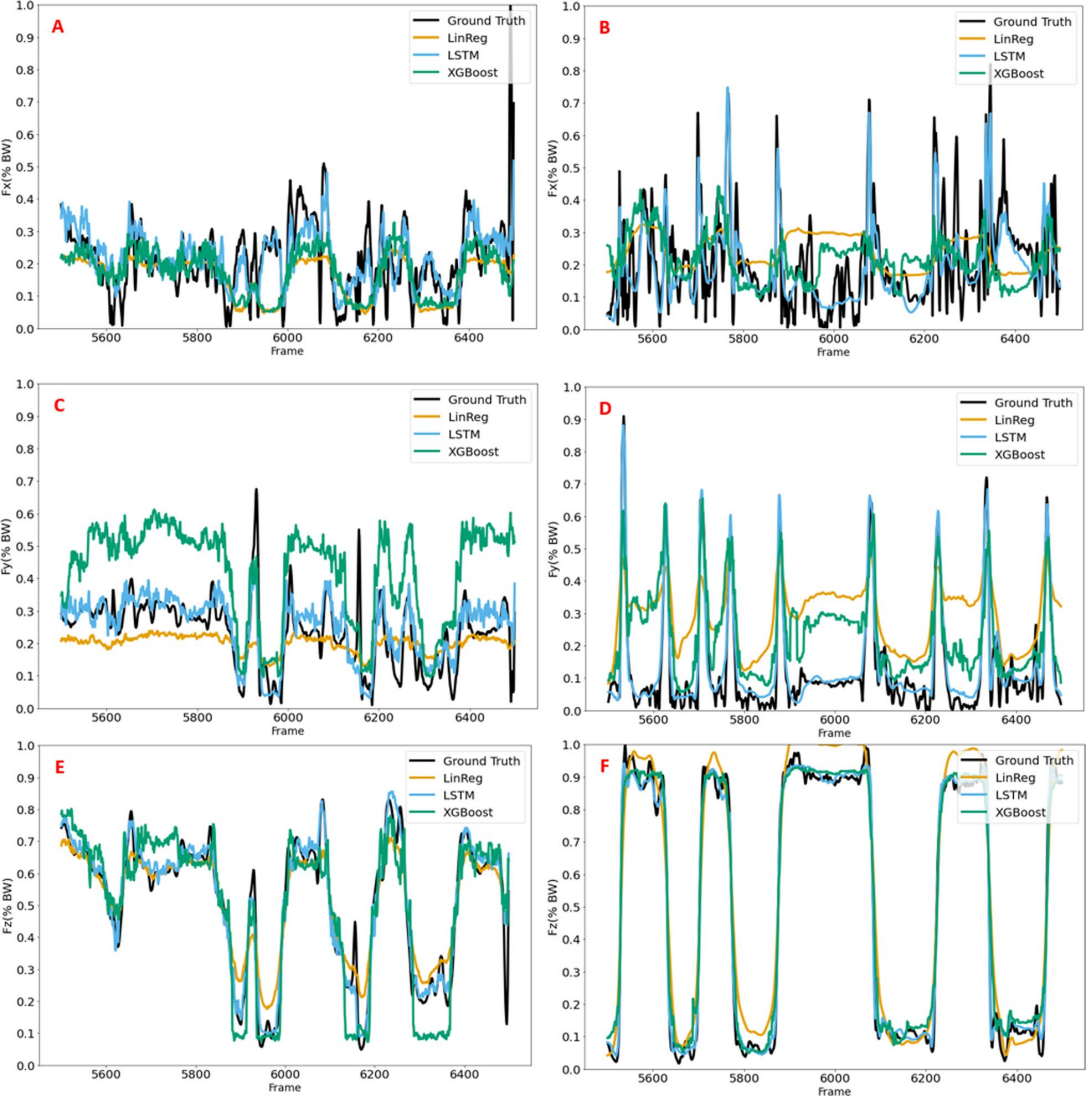
Example of estimation performance on the left side from XGBoost (green), LSTM (blue), and LinReg (orange) models in each GRF component over 1000 frames, along with the ground truth GRF (black). **A**, **C**, and **E** Results from one 2D-DV dataset, and **B**, **D** and **F** One MoCap dataset

In Fig. 8, example plots from the left foot are presented that show the estimated component values by the XGBoost, LSTM, and LinReg models over a random set of 1000 frames, along with the ground truth component values. The LSTM model estimates all three components very well, both using MoCap and 2D-DV data. The $F_x$ component seems to be the least accurate, although the LSTM model estimates the major changes in BW here as well. The XGBoost model also estimates $F_z$ very well, but this is not seen to the same degree in $F_y$ and $F_x$. In $F_x$ and $F_y$ the XGBoost model is able to follow the major trends in the data, but rapid changes in force are not estimated well. The LinReg model is able to estimate major changes in $F_z$, but not with the level of detail seen in the LSTM or XGBoost model. $F_x$ and $F_y$ components, however, are not estimated as well by the LinReg model.

### Test/train error

**Fig. 9:**
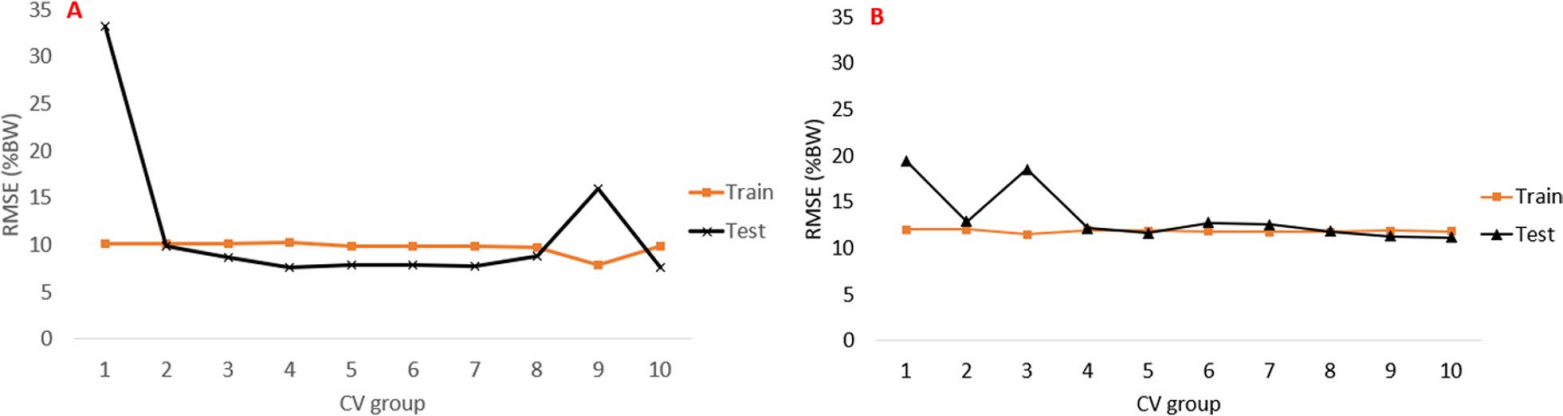
XGBoost model test/train RMSE (%BW) from each cross-validation iteration using 3DMoCap and 2D-DV data

**Fig. 10:**
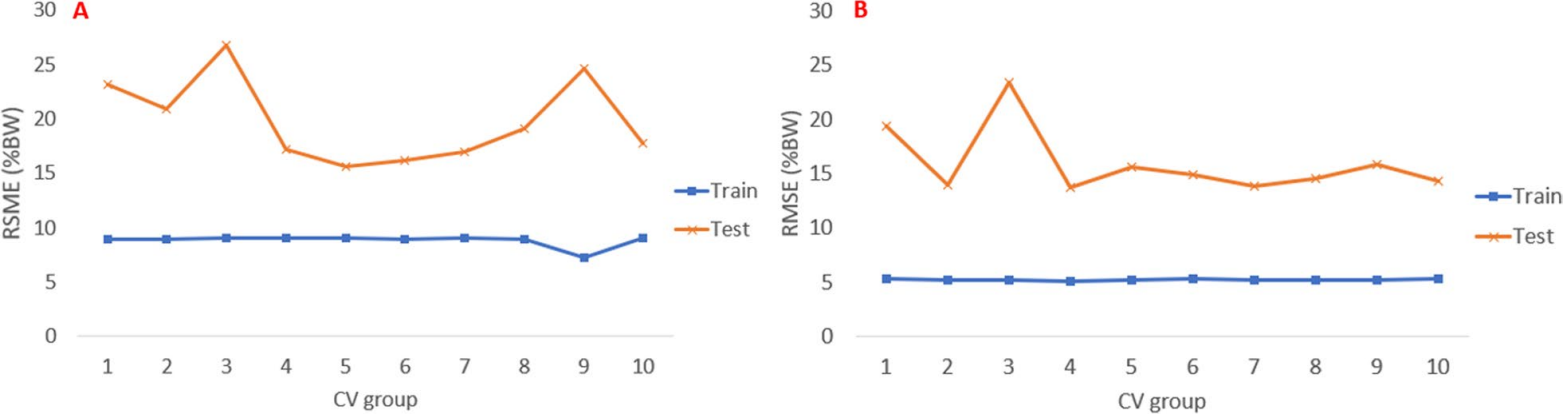
LSTM model test/train RMSE (%BW) from each cross-validation iteration using 2D-DV (**A**) and 3DMoCap (**B**) data

The LSTM and XGBoost RMSE from using test and train data is presented in Figs. 9 and 10, respectively. The test error for the XGBoost model is consistently about 3$\times$ higher than the train error, using both 3DMoCap and 2D-DV data. For XGBoost, the mean (±SD) train/test RMSE was 8.8 (.5)/19.8 (3.7) %BW, respectively, using 2D-DV data and 5.2 (.1)/15.9 (3.0) %BW, respectively, using 3DMoCap data. For LSTM, the mean (±SD) train/test RMSE using 2D-DV data was 9.8 (.6)/11.5 (7.6) %BW, respectively, and 11.85 (.2)/13.6 (2.9) %BW, respectively, using 3DMoCap data.

## Discussion

This study investigated two facets of estimation of GRF components in balance training using machine learning models. First, we assessed the overall estimation performance of an LSTM and an XGBoost model on GRF components, comparing it to a baseline LinReg model’s performance. Second, the performance of the LSTM and XGBoost models in estimating 3D GRF data using 2D joint data was examined. Overall, the LSTM model performance was very good, considering that joint position data was the only input data used for estimation. The LSTM RMSE was $< 11$% BW for all GRF components when using 3DMoCap data, and $R^2$ was moderate to high ($> .58$ and $> .79$) for $F_x$ and $F_y$, and excellent ($> .97$) for $F_z$. This shows that the LSTM model was able to accurately estimate the $F_z$ component, while achieving only slightly less accurate results in the $F_x$ and $F_y$ components. The boxplots in Fig. 7 also show that the $F_z$ estimation was very stable around the median. This was the case in all three models.

The most promising part of our results is that our method does not require information about the person playing or any calculations using the input data to represent the person—i.e., no biomechanical model is needed. This makes our method less computationally expensive, and easier to implement in an in-home setting. Still, our findings on estimation of GRF from kinematic data are in line with related literature in gait analysis, such as Mundt et al. [16], Oh et al. [17], and Choi et al. [18]. The movement pattern is different, so a direct comparison of results is not feasible. These studies used 3DMoCap data to calculate biomechanical features such as joint angles [17, 36], body segment velocities [18], and foot contact events [21], which are not obtainable using only joint position data. This demonstrates the strength in our results: our method use the joint center positions directly, skipping both practical and computational steps that complicate the process. This makes our method more accessible and easy to use, while being as accurate as more complicated methods.

Regarding performance using 2D-DV data, our findings support using this modality for estimation of $F_z$ during balance exergaming. This is a step in the right direction regarding in-home use of exergaming, as a standard digital camera that most people already possess can provide accurate information about weight shifting performance during exergaming. This can be achieved in the form of a smartphone or a web camera instead of needing to acquire a specialized device such as a Kinect camera. However, our findings also show that when the context requires three dimensional GRF data, the use of 3D kinematic data is preferred to ensure estimation accuracy in all three GRF components. This is also true when the context requires model performance that has a $< 10$% BW error requirement in other components than $F_z$.

LinReg also performs surprisingly well in $F_z$, with comparable RMSE and $R^2$ to LSTM and XGBoost, although both the LSTM and XGBoost models are better at estimating the small changes in force that occurs between lateral weight-shifts (i.e., when the person is standing with the majority of their BW on one foot).

The $F_z$ component is arguably the most informative of the three directions in balance training, as it represents the vertical force—i.e., the weight that is being pushed straight downwards onto the surface. In practice, this informs about how much body weight the person places on each leg, which is an indication of how well the person is performing a weight shift during exercise. However, $F_x$ and $F_y$ information may also be relevant to measure accurately as the force exerted in these directions contribute to postural control. For example, force magnitude, directional accuracy, and variability in $F_y$ and $F_x$ in relation to a (externally or internally induced) disturbance in posture can be informative about balance ability [37, 38]. In medio-lateral weight-shifting the $F_x$ component might not be as critical to measure as the $F_z$ component measures the same movement in this context. In contrast, control over anterior–posterior movement (and thus $F_y$) is important to maintaining a steady and stable sideways movement pattern, to prevent large anterior–posterior movements during weight-shifting exercises and potentially create destabilizing conditions. This means that even though $F_z$ provides the main information about sideways weight-shifting performance, $F_y$ can inform about the variability and stability in a weight-shifting movement.

The feature importance information from the XGBoost model showed different joints to be important based on the type of data used. When using 3D data, more joints from the right side contributed to estimation performance, while more joint on the left side were important when using 2D data. From these results we were not able to elucidate any systematic or clear pattern in joint importance, which might be caused by the limited set of movements performed in this study. This might be an interesting avenue to explore further using a data set richer in terms of movements.

The high $R^2$ achieved could be a sign of overfitting by the LSTM model [39]. However, the tenfold CV process showed a stable fit using test data, which can be seen in the low spread of the LSTM model in Fig. 7 as well. Results from test/train errors also support this, as the difference between test/train errors is low, as seen in Fig. 10. Even more reassuring is the fact that the CV process was not a holdout of random pieces of data, but a holdout of all the data from each person. Thus, estimation of GRF was performed on previously unseen data from a person with an unknown movement pattern.

The XGBoost model, however, does indeed seem to suffer from overfitting, which presents itself as higher RMSE when estimating based on unseen data compared to training data [40] (Fig. 9). This is likely caused by either too much noise in the data (especially in the 2D-DV data), where the limited tree depth (max depth = 12) does not allow for the tree to fully model the real relationship in the data, or that the current data set is too sparse. Even though XGBoost inherently possesses features that are known to prevent overfitting, our findings indicate that this was not successful here.

## Limitations

There are some limitations to be aware of in the current study. The movement pattern performed by participants was limited to to sideways leaning, and there were a low number of participants. The data was collected in a laboratory setting, and the models used require training data to be usable in a real-world setting.

## Conclusion

In conclusion, the LSTM model performed very well, especially in $F_z$. 3DMoCap data produced the best results, and the best $F_z$ estimation from 2D video data is also achieved by using the LSTM model. These findings show that it is feasible to develop exergames that provides weight-shifting biofeedback by only using 2D joint position data from a standard digital video camera. With the support of a standard camera, an exergame in balance training can incorporate the LSTM model to provide real-time biofeedback on weight-shifting performance. This warrants further investigation into how such systems can be integrated into exergames for in-home or in balance exercise, as it opens up broad opportunities for providing accurate feedback in a simple, yet accurate manner. The LSTM model and 2D-DV input data combination has the potential to facilitate more effective and motivating in-home balance training by incorporating accurate feedback on weight-shifting performance in exergames.

## Data Availability

The data set used and/or analysed during the current study are available from the corresponding author on reasonable request.

## Abbreviations

2D: Two-dimensional
2D-DV: Digital video
3DMoCap: Three-dimensional motion capture
AI: Artificial intelligence
BW: Body weight
CV: Cross-validation
DL: Deep learning
DLC: DeepLabCut
GRF: Ground reaction force
IMU: Inertial measurement unit
LinReg: Linear regression
LSTM: Long short-term memory
ML: Machine learning
PiG-FB: Plug-in-Gait Full Body
$R^2$: Coefficient of determination
RMSE: Root mean square error
SD: Standard deviation
XGBoost: Extremely boosted gradient trees
